# Neuroprotective Effect of Astragaloside IV on Cerebral Ischemia/Reperfusion Injury Rats Through Sirt1/Mapt Pathway

**DOI:** 10.3389/fphar.2021.639898

**Published:** 2021-03-26

**Authors:** Yi-Hua Shi, Xi-Le Zhang, Peng-Jie Ying, Zi-Qian Wu, Le-Le Lin, Wei Chen, Guo-Qing Zheng, Wen-Zong Zhu

**Affiliations:** ^1^ Department of Neurology, The Second Affiliated Hospital and Yuying Children's Hospital of Wenzhou Medical University, Wenzhou, China; ^2^ Department of Neurology, Wenzhou Hospital of Traditional Chinese Medicine Affiliated toZhejiang Chinese Medical University, Wenzhou, China; ^3^ Department of Radiology, The Second Affiliated Hospital and Yuying Children’s Hospital of Wenzhou Medical University, Wenzhou, China

**Keywords:** traditional Chinese medicine, ischemic stroke, silent information regulator 1, phosphorylation, acetylation

## Abstract

**Background:** Ischemic stroke is a common disease with poor prognosis, which has become one of the leading causes of morbidity and mortality worldwide. Astragaloside IV (AS-IV) is the main bioactive ingredient of Astragali Radix (which has been used for ischemic stroke for thousands of years) and has been found to have multiple bioactivities in the nervous system. In the present study, we aimed to explore the neuroprotective effects of AS-IV in rats with cerebral ischemia/reperfusion (CIR) injury targeting the Sirt1/Mapt pathway.

**Methods:** Sprague–Dawley rats (male, 250–280 g) were randomly divided into the Sham group, middle cerebral artery occlusion/reperfusion (MCAO/R) group, AS-IV group, MCAO/R + EX527 (SIRT1-specific inhibitor) group, and AS-IV + EX527 group. Each group was further assigned into several subgroups according to ischemic time (6 h, 1 d, 3 d, and 7 days). The CIR injury was induced in MCAO/R group, AS-IV group, MCAO/R + EX527 group, and AS-IV + EX527 group by MCAO surgery in accordance with the modified Zea Longa criteria. Modified Neurological Severity Scores (mNSS) were used to evaluate the neurological deficits; TTC (2,3,5-triphenyltetrazolium chloride) staining was used to detect cerebral infarction area; Western Blot was used to assess the protein levels of SIRT1, acetylated MAPT (ac-MAPT), phosphorylated MAPT (*p*-MAPT), and total MAPT (t-MAPT); Real-time Quantitative Polymerase Chain Reaction (qRT-PCR) was used in the detection of Sirt1 and Mapt transcriptions.

**Results:** Compared with the MCAO/R group, AS-IV can significantly improve the neurological dysfunction (*p* < 0.05), reduce the infarction area (*p* < 0.05), raise the expression of SIRT1 (*p* < 0.05), and alleviate the abnormal hyperacetylation and hyperphosphorylation of MAPT (*p* < 0.05). While compared with the AS-IV group, AS-IV + EX527 group showed higher mNSS scores (*p* < 0.05), more severe cerebral infarction (*p* < 0.05), lower SIRT1 expression (*p* < 0.01), and higher ac-MAPT and *p*-MAPT levels (*p* < 0.05).

**Conclusion:** AS-IV can improve the neurological deficit after CIR injury in rats and reduce the cerebral infarction area, which exerts neuroprotective effects probably through the Sirt1/Mapt pathway.

## Highlights

•The important role of MAPT in neurological diseases has been paid more and more attention. The present study preliminarily investigated the post-translational modifications of MAPT in ischemic stroke.

•The present study preliminarily showed that regulating SIRT1 can further affect MAPT protein, providing a new idea to the pathogenesis of cerebral ischemia.

•In this study, a new possible mechanism of Astragaloside IV in cerebral ischemia model was studied, which provided a new approach to the study of therapy for cerebrovascular diseases.

## Introduction

Ischemic stroke has become one of the leading causes of death and acquired disability world widely (James et al., 2018). Over time after stroke, the degree of neuron death and synapse loss increases exponentially (Radak et al., 2017). Thus, early intervention after symptom onset is important to improve the prognosis of ischemic stroke. Intravenous thrombolysis and mechanical thrombectomy are effective therapies according to the 2018 guideline from the American Heart Association/American Stroke Association (AHA/ASA) (Khatri et al., 2018; Powers et al., 2019). However, the usage of recombinant tissue plasminogen activator (rtPA) still remains some limitations, such as the 4.5 h treatment time window and the risk of hemorrhage (Yaghi et al., 2017; Kim, 2019). Mechanical thrombectomy should be performed within 24 h, requiring trained vascular surgeons and specialized stroke centers, which limits its universality (Moussaddy et al., 2018). In addition, even after successful interventions, a lot of ischemic stroke patients still have neurological dysfunction, seriously affecting their life quality (Grabowska-Fudala et al., 2018). Thus, it is urgent to explore novel treatment options of ischemic stroke.

Silent information regulator 1 (SIRT1), a class III histone deacetylase, is widely expressed in a variety of cell types, and affects the physiological activities of cells by regulating the acetylation and deacetylation status of proteins (Imai et al., 2000; Kitajewski, 2011). Previous studies have found that the overexpression of SIRT1 significantly reduces brain damage in mice with ischemic stroke (Hellberg et al., 2010; Hernandez-Jimenez et al., 2013), probably through anti-inflammation (Wiciński et al., 2018), anti-apoptosis (Yang et al., 2015), anti-oxidation (Lv et al., 2015), regulation of metabolism (Koronowski et al., 2017), and promotion of neural regeneration (Zhao et al., 2015). Recently, it has also been found that SIRT1 is closely related to microtubule-associated protein tau (MAPT) modification (Min et al., 2010; Min et al., 2018).

MAPT is mainly distributed in neurons of central nervous system (Avila et al., 2004). The post-translational modification of MAPT helps the binding of tubulin and the stability of microtubule and neuronal morphology (Avila et al., 2004). However, when the modifications of MAPT are unbalanced, such as abnormally highly phosphorylation, the binding capacity of microtubules will reduce, leading to a decrease in the stability of the neuronal cytoskeleton, and ultimately death of the neurons (Min et al., 2010; Cohen et al., 2011). Studies have revealed that a large number of abnormal hyperphosphorylated MAPT exists in cerebral ischemia/reperfusion (CIR) animal models, which is closely related to neuronal apoptosis and neurological deficits (Basurto-Islas et al., 2018). Lately, Min et al. (2018) found that hyperacetylation of MAPT can affect other post-translational modifications, such as reducing the degradation of phosphorylated MAPT and causing its accumulation. In addition, it has also been found that SIRT1 deacetylates MAPT at specific lysine residues (probably at positions 160–182 and 264–287) (Min et al., 2010; Min et al., 2018). These evidences suggested that modification of Sirt1/Mapt pathway may play a critical role during stroke recovery.

**FIGURE 1:**
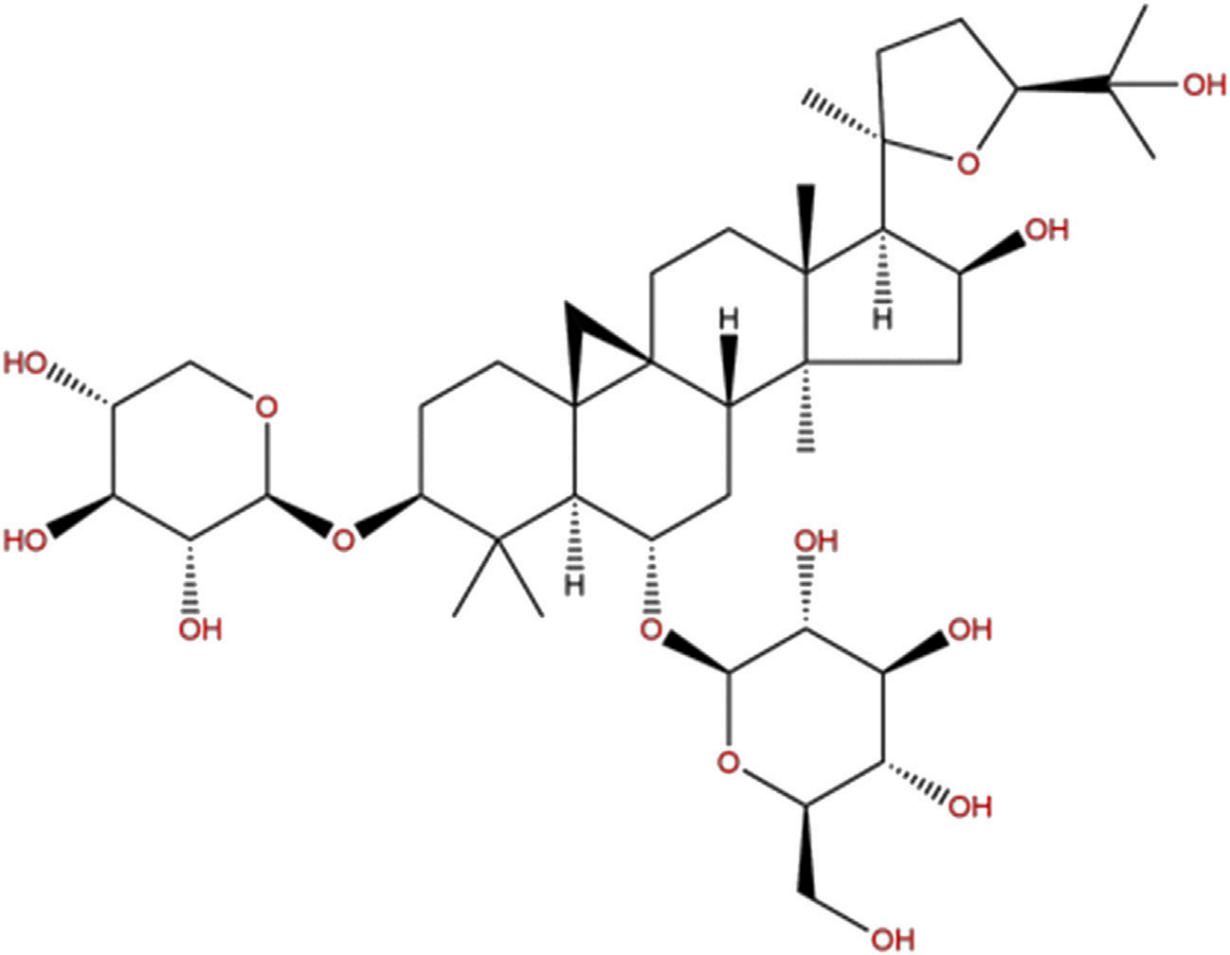
The chemical structure of AS-IV.

Chinese herbal medicine has been applied in the treatment of cerebral ischemia for thousands of years and has accumulated a lot of clinical experience (Li et al., 2016; Seto et al., 2016). Astragali Radix is the dried root of the perennial leguminous plant *Mongolian Astragalus* or *Astragalus membranaceus* which was first recorded in the Shennong Materia Medica (220–280 AD) (Gong et al., 2018) and was used for ischemic stroke since Tang Dynasty. Pharmacological studies have found that Astragali Radix contains a variety of bioactive substances, including saponins, polysaccharides, flavonoids, amino acids, and trace elements (Fu et al., 2014), of which Astragaloside IV (AS-IV, chemical formula C_41_H_68_O_14_, the chemical structure is shown in Figure 1) is the main active ingredient and is included in the Pharmacopoeia of China (2015 edition) as a quality control marker (National, 2015). Previous studies have shown that AS-IV has a variety of biological activities such as anti-apoptosis (Wang et al., 2020), anti-inflammation (Chen et al., 2020), anti-oxidation (Wang et al., 2019), vasodilation (Zhang et al., 2006), and improving microcirculation perfusion (Yu et al., 2015). Our previous pre-clinical systematic reviews also indicated that AS-IV exerts a neuroprotective effect in CIR animals (Wang et al., 2017), but the mechanisms remain unclear. Thus, the present study was aimed to investigate the neuroprotective effects of AS-IV in rats after CIR injury through Sirt1/Mapt T pathway.

## Materials and Methods

### Ethics Statement

All experimental subjects were obtained from the Beijing Weitong Lihua Experimental Animal Technology Co., Ltd. (License number: SCXK(Jing)2016-0011). The protocol was approved by the local ethics committee of Wenzhou Medical University (License number: wydw2015-0148) and was performed in strict accordance with its guidelines. There were no restrictions on activities, feeding and drinking of the subjects. All subjects received anesthesia at the end of experiment to minimize animal suffering, and the utmost efforts were made to reduce the number of experimental animals.

### Animals and Groups

Adult male Sprague-Dawley (SD) rats were raised separately with five in each cage. Keeping the temperature at 21–25°C, the relative humidity at around 50%, and a 12 h light/dark cycle. After a week of adaptation to the experimenter (when the body weights were around 250–280 g), SD rats were randomly divided into 5 groups: Sham group, middle cerebral artery occlusion/reperfusion (MCAO/R) group, AS-IV group, MCAO/R + EX527 group, and AS-IV + EX527 group. The Sham group, MCAO/R group, and AS-IV group were further divided into four subgroups at 6 h, 1 d, 3 d, and 7 days after CIR injury, respectively. EX527 intervened groups and their control groups were monitored only at 7 days after CIR injury. There were 12 rats in each subgroup.

### Drug Administration

AS-IV was obtained from Shanghai Tongtian Biotechnology Co., Ltd. (No. 84687-43-4, purity ≥98%), and was dissolved in normal saline fully. The rats in AS-IV group and AS-IV + EX527 group received AS-IV intraperitoneally at a dose of 20 mg/(kgd) (Qu et al., 2009; Li et al., 2020). The Sham group, MCAO/R group and MCAO/R + EX527 group received same volume of saline intraperitoneally instead. All subjects were dosed once a day from 3 days before the operation to the end of the experiment. EX527 is a SIRT1-specific inhibitor acquired from Selleck Chemicals (No. S1541, purity: 99.78%) and dissolved in DMSO fully. Intracerebroventricular injection of EX527 at a dose of 10 µg per 2 days was performed to the rats in AS-IV + EX527 group and MCAO/R + EX527 group from 3 days before the operation until the rats were sacrificed. Similarly, the control groups without intervention of EX527 were set up. The Sham group, the MCAO/R group and the AS-IV group all underwent the same operation, and the same volume of DMSO was injected.

### Animal Models

After anesthesia with 10% chloralhydrate (3 ml/kg), the rats were operated on according to the modified Zea Longa method (Longa et al., 1989) to induce CIR injury. In brief, a midline incision was performed on neck and the left common carotid artery (CCA), internal carotid artery (ICA), and external carotid artery (ECA) were gently isolated. Then, a monofilament nylon suture with a rounded tip (diameter: 0.36 ± 0.02 mm, lot: 2636-A5, Beijing Cinontech Co., Ltd., China) was introduced into the ECA and slowly inserted into the ICA to block the origin of the middle cerebral artery (MCA). After 2 h of occlusion, the nylon suture was removed to establish reperfusion. As for the Sham group, all the rats received the same surgery without the occlusion of MCA. The rectal temperature of the rats was maintained at 37 ± 0.5°C throughout the surgical procedure. After waking from the anesthesia, the rats were put back to cages with free food and water.

Rats were examined for the neurological deficiency preliminary at 2 h after MCAO according to Longa’s five-tiered grading system as follows: 0, no deficit; 1, failure to extend contralateral forepaw; 2, spin longitudinally; 3, falling to the contralateral side; 4, unable to walk spontaneously. Only rats with a score between 1 and 3 were considered successful models and then included in subsequent researches.

### Neurological Deficiency Score

Assessment of the neurological deficiency score (NDS) was performed at 6 h, 1 d, 3 d, and 7 days after CIR injury by an investigator who was blind to the grouping. From the four aspects of movement, sensation, balance and reflex, the assessment was carried out strictly according to the modified neurological severity score (mNSS) criteria (Chen et al., 2001; Wang et al., 2015) as follows: 0, no deficit; 1–6, mild deficit; 7–12, moderate deficit; 13–18, severe deficit. The specific scoring rules are shown in Table 1.

**TABLE 1:** Modified Neurological Severity Score criteria.

Items	Scores
Motor tests	6
Raising rat by tail	3
Flexion of forelimb	1
Flexion of hindlimb	1
Head moved >10° to vertical axis within 30 s	1
Placing rat on floor (normal = 0, maximum = 3)	3
Normal walk	0
Inability to walk straight	1
Circling toward paretic side	2
Falls down to paretic side	3
Sensory tests	2
Placing test (visual and tactile test)	1
Proprioceptive test (deep sensation, pushing paw against table edge to stimulate limb muscles)	1
Beam balance tests (normal = 0; maximum = 6)	6
Balances with steady posture	0
Grasps side of beam	1
Hugs beam and one limb fall down from beam	2
Hugs beam and two limbs fall down from beam, or spins on beam (>60 s)	3
Attempts to balance on beam but falls off (>40 s)	4
Attempts to balance on beam but falls off (>20 s)	5
Falls off; no attempt to balance or hang on to beam (<20 s)	6
Reflex absence and abnormal movements	4
Pinna reflex (head shake when auditory meatus is touched)	1
Corneal reflex (eye blink when cornea is lightly touched with cotton)	1
Startle reflex (motor response to a brief noise from snapping a clipboard paper)	1
Seizures, myoclonus, myodystony	1
Maximum points	18

Grade: 0, no deficit; 1–6, mild deficit; 7–12, moderate deficit; 13–18, severe deficit.

### Cerebral Infarction Area

At each time points, rats were deeply anesthetized with 10% chloralhydrate (300 ml/kg) and thoroughly perfused with saline. Brains were removed quickly, excised olfactory bulb, cerebellum and brainstem, and were sectioned coronally at 2 mm intervals from the frontal pole. The slices were then immersed in 1% triphenyl tetrazolium chloride (TTC, Sigma) solution for 20 min at 37°C in the dark, and then fixed with 4% paraformaldehyde at 4°C for 24 h. The un-infarcted part in the brain slice was red and the infarcted part was white. Image-Pro Plus 6.0 software was used to calculate the infarction area and total area of brain slice.

### Western Blot

Brain tissues from ipsilateral hippocampus were lysed in RIPA buffer (Beyotime, China) containing PMSF (Beyotime, China) and phosphatase inhibitor (Solarbio, China). The total protein concentration in supernatant was determined by a BCA protein assay kit (Beyotime, China). Aliquots of homogenate with equal protein concentration were separated by 8% SDS-PAGE gel and then transferred to polyvinyl difluoramine membrane. After blocking in 5% non-fat milk for 2 h at room temperature, the membranes were then incubated with primary antibodies, including rabbit anti-SIRT1 (1:1000, D1D7, CST), mouse anti-tau (tau-5, 1:1000, ab80579, abcam), rabbit anti-tau (phospho-S396, 1:1,000, ab109390, abcam), rabbit anti-tau (acetyl-Lys174, 1:1000, ABP60620, Abbkine), or mouse anti-GAPDH (1:2500, TA802519, OriGene) at 4°C overnight. After washing by TBST for 3 times, the blots were then incubated with anti-rabbit or anti-mouse IgG conjugated to HRP for 1.5 h at room temperature and visualized using the enhanced chemiluminescence.

### Real-Time Quantitative Reverse Transcription Polymerase Chain Reaction (RT-qPCR)

Total RNA was extracted using Trizol reagent (Invitrogen, United States). Then, the RNA of each group was reverse transcribed into cDNA with PrimeScriptTM RT reagent Kit (TAKARA, RR047A, Japan). Quantitative RT-qPCR was performed by a Light Cycler thermal cycler system (Bio-Rad, United States) using iQ SYBR Green Supermix (Bio-Rad, United States). The designed gene-specific primers were as follows: Sirt1: forward, 5′-GAG​TGT​GCT​GGA​GGA​TCT​G -3′, and reverse, 5′- TGC​TCT​GAT​TTG​TCT​GGT​GT-3′; total Mapt: forward, 5′-CCA​GGA​GTT​TGA​CAC​AAT​GGA​AGA​C-3′, and reverse, 5′-CTG​CTT​CTT​CAG​CTT​TTA​AGC​CAT​G-3′; GAPDH: forward, 5′-ATG​GCT​ACA​GCA​ACA​GGG​T-3′, and reverse, 5′-TTA​TGG​GGT​CTG​GGA​TGG-3′.

### Statistical Analysis

Graph Pad Prism 8.0 (Graph Pad, United States) was used for statistical analyses and all the results were expressed as means ± standard deviation (mean ± SD). One-way analysis of variance (ANOVA) was used to analyze differences between multiple groups, while Tukey test was used to analyze differences between two groups. Values of *p* < 0.05 were considered statistically significant.

## Results

### Effect of Astragaloside IV on Neurological Deficits

**FIGURE 2:**
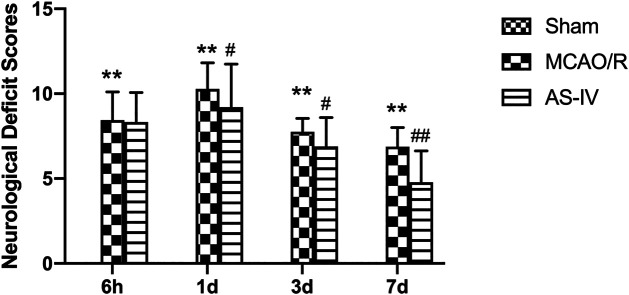
The neurological deficit scores (NDS) in Sham group, middle cerebral artery occlusion/reperfusion (MCAO/R) group and Astragaloside IV (AS-IV) group at 6 h, 1 d, 3 d, and 7 days after CIR injury in rats (mean ± SD, *n* = 12). **p < 0.01, compared with the Sham group; #*p* < 0.05, ##*p* < 0.01, compared with the MCAO/R group.

CIR injury involves multiple neurological disorders, such as motor and sensory dysfunction. In the present study, the NDS was evaluated at 6 h, 1 d, 3 d, and 7 days after CIR according to mNSS criteria. The higher the score, the more severe the dysfunction. No sign of neurological deficits was detected in the Sham group. The MCAO/R group had obvious symptoms of neurological deficits compared with the Sham group (*p* < 0.01), specifically, NDS increased at 6 h after reperfusion, peaked at 1 day, and then decreased gradually. Compared with the MCAO/R group, the AS-IV group showed significantly lower NDS at 1 d, 3 d, and 7 days after CIR injury (*p* < 0.05) (Figure 2).

### Effect of Astragaloside IV on Cerebral Infarction Area

**FIGURE 3:**
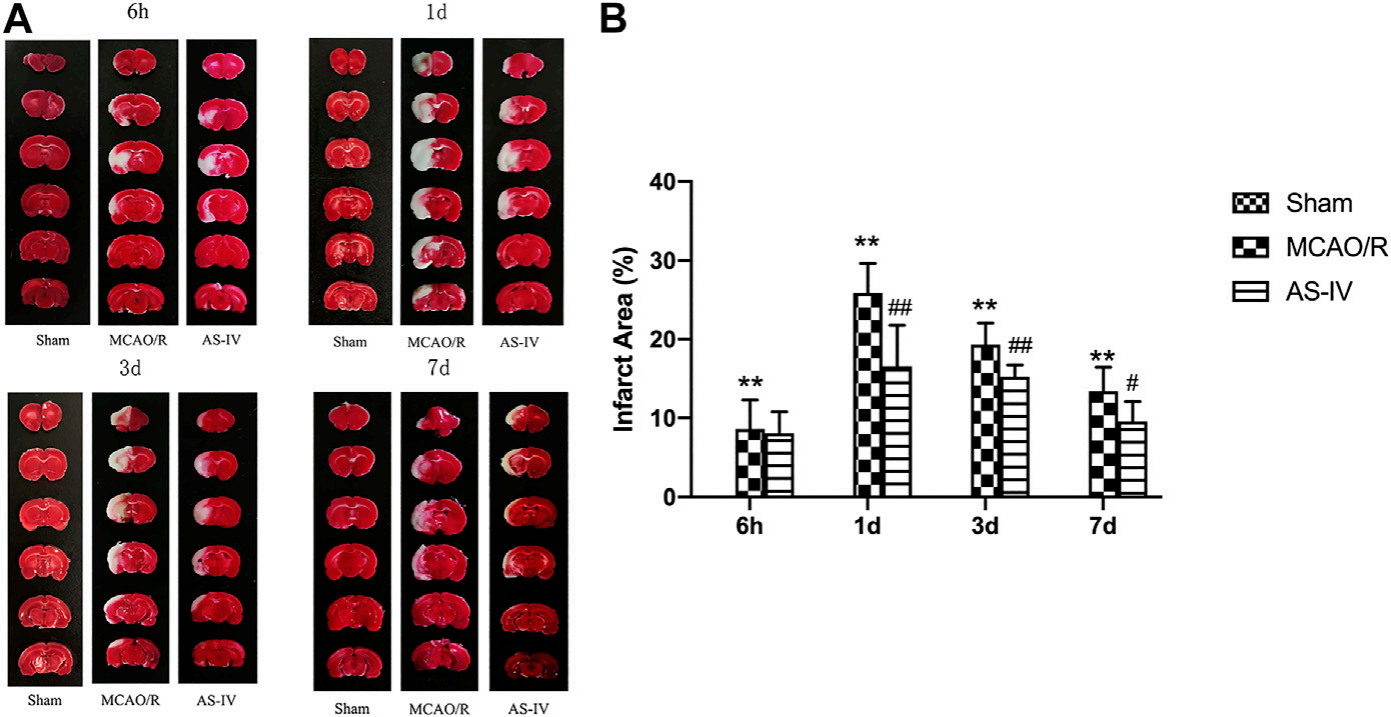
The cerebral infarction areas in Sham group, MCAO/R group and AS-IV group at 6 h, 1 d, 3 d, and 7 days after CIR injury in rats (mean ± SD, *n* = 6). **(A)** TTC staining **(B)** Quantitative analysis for the percentage of the infarction area. **p < 0.01, compared with the Sham group; #*p* < 0.05, ##*p* < 0.01, compared with the MCAO/R group.

TTC staining illustrated that there was no cerebral infarction in the Sham group. Compared with the Sham group, significant infarction area could already be detected in MCAO/R group at 6 h after reperfusion, reached a peak at 1 day, and then declined gradually (*p* < 0.01). Compared with the MCAO/R group, AS-IV significantly reduced infarction areas at 1 d, 3 d, and 7 days after CIR injury (*p* < 0.05) (Figure 3).

### Effect of Astragaloside IV on the Expression of SIRT1

**FIGURE 4:**
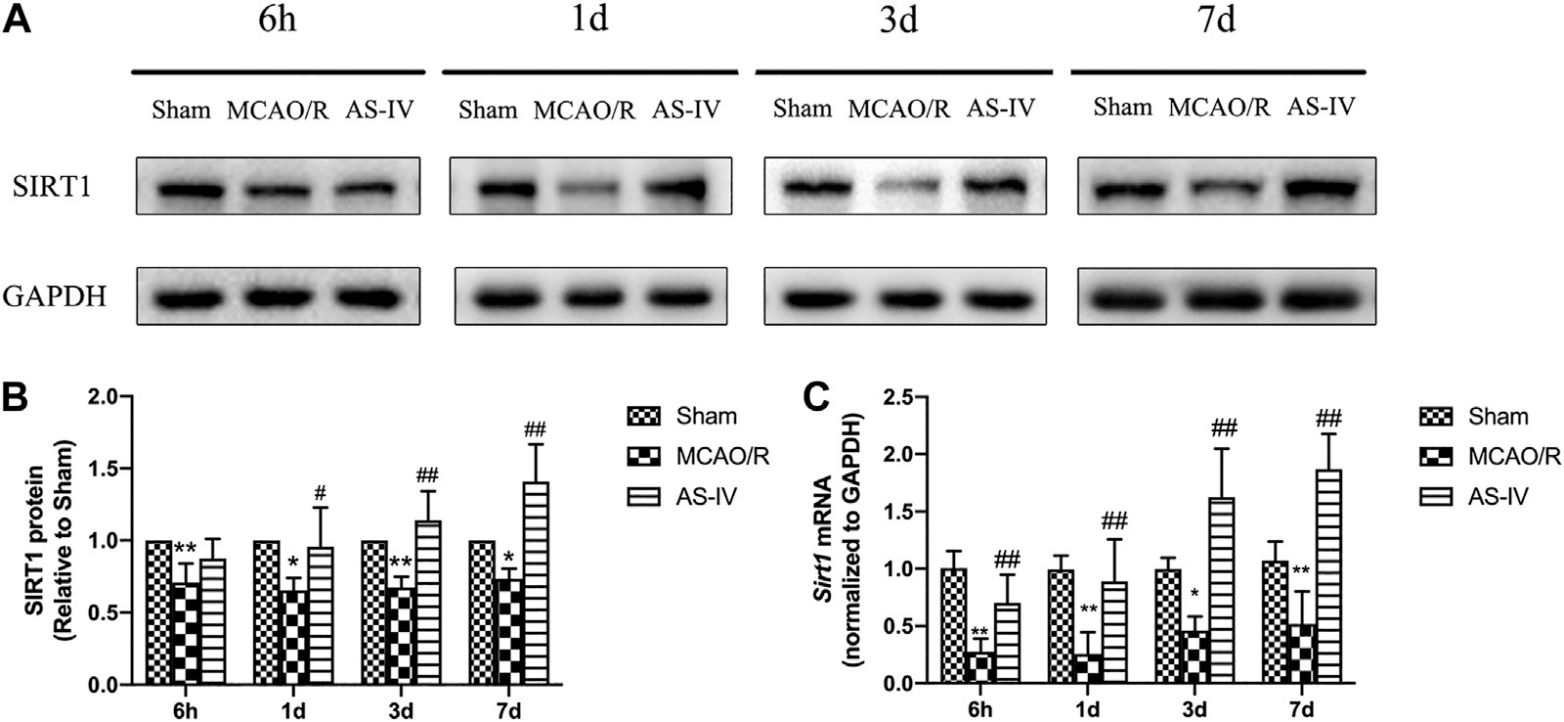
The expression of SIRT1 in Sham group, MCAO/R group, and AS-IV group at 6 h, 1 d, 3 d, and 7 days after CIR injury (mean ± SD, *n* = 6). **(A)** The results from the Western Blot. **(B)** Quantitative analysis for the protein level of SIRT1. **(C)** Quantitative analysis for the mRNA level of Sirt1. *p < 0.05, **p < 0.01, compared with the Sham group; #*p* < 0.05, ##*p* < 0.01, compared with the MCAO/R group.

Western Blot (Figures 4A,B) and RT-qPCR (Figure 4C) revealed that the expression of SIRT1 had no significant difference in Sham group among the time point of 6 h, 1 d, 3 d, and 7 days. After CIR injury, SIRT1 protein and mRNA levels significantly decreased at any time point compared with the Sham group (*p* < 0.05). Compared with the MCAO/R group, AS-IV significantly increased the SIRT1 protein level at 1 d, 3 d, and 7 days (*p* < 0.05), and in mRNA level at each time point (*p* < 0.01).

### Effect of Astragaloside IV on the Expression of MAPT

**FIGURE 5:**
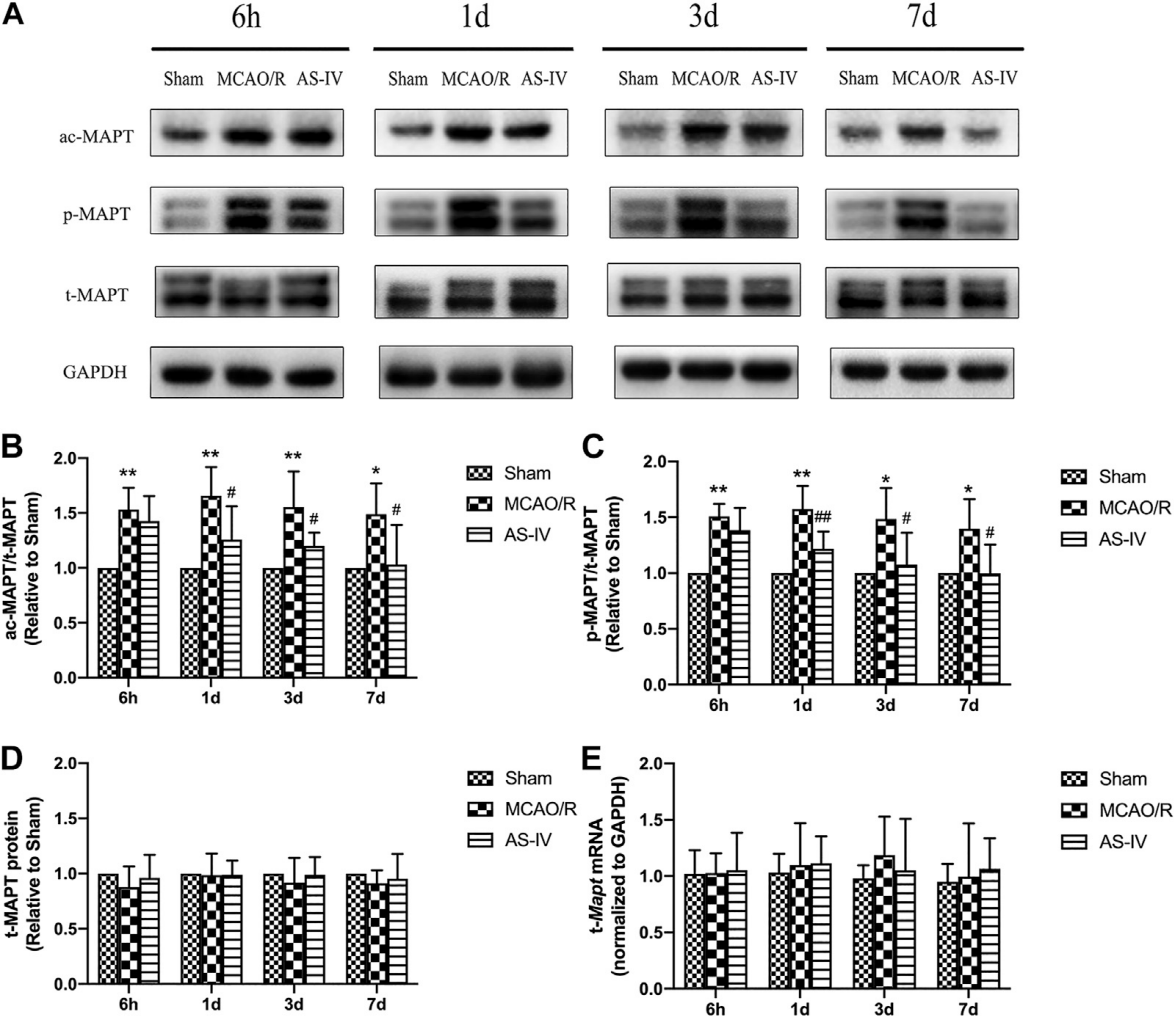
The expressions of total MAPT (t-MAPT), acetylized MAPT (ac-MAPT), and phosphorylated MAPT (*p*-MAPT) in Sham group, MCAO/R group, and AS-IV group at 6 h, 1 d, 3 d, and 7 days after CIR injury (mean ± SD, *n* = 6). **(A)** The results from the Western Blot. **(B)** Quantitative analysis for the protein level of ac-MAPT. **(C)** Quantitative analysis for the protein level of *p*-MAPT **(D)** Quantitative analysis for the protein level of t-MAPT. **(E)** Quantitative analysis for the mRNA level of t-Mapt. *p < 0.05, **p < 0.01, compared with the Sham group; #*p* < 0.05, ##*p* < 0.01, compared with the MCAO/R group.

We evaluated the expressions of total MAPT (t-MAPT), acetylized tau (ac-MAPT), and phosphorylated tau (*p*-MAPT) by Western Blot and RT-qPCR. The present results showed that low-level ac-MAPT and *p*-MAPT existed in the Sham group (Figure 5A). Compared with the Sham group, the levels of ac-MAPT and *p*-MAPT obviously increased at each time point after CIR injury (*p* < 0.05). And compared with the MCAO/R group, AS-IV significantly decreased the expression of ac-MAPT (Figures 5A,B) and *p*-MAPT (Figures 5A,C) at 1 d, 3 d, and 7 days (*p* < 0.05). The protein and mRNA levels of t-MAPT (Figures 5A,D,E) had no significant differences comparing AS-IV group with Sham or MCAO/R groups among all the time point.

### Effects of EX527 on Neurological Deficiency Score Intervened by Astragaloside IV

**FIGURE 6:**
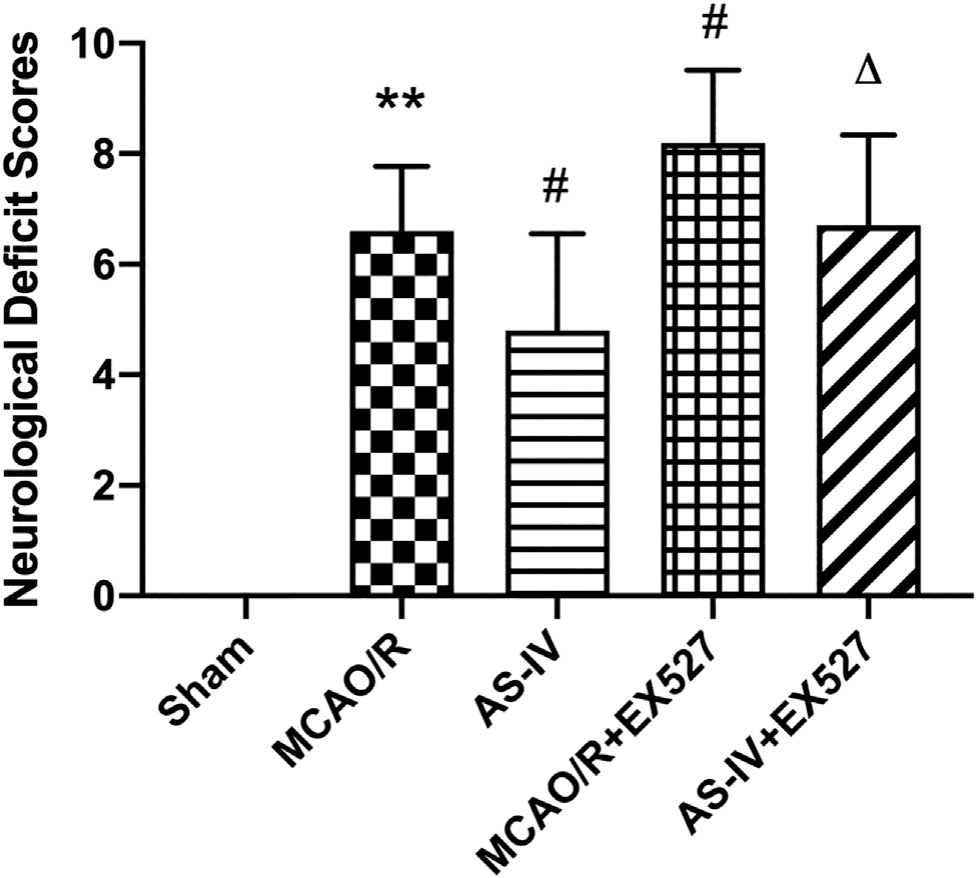
The NDSs in Sham, MCAO/R, AS-IV, MCAO/R + EX527, and AS-IV + EX527 group at 7 days after CIR injury in rats (mean ± SD, *n* = 12). **p < 0.01, compared with the Sham group; #*p* < 0.05, compared with the MCAO/R group; ∆*p* < 0.05 compared with the AS-IV group.

The above results indicated that AS-IV can improved neurological deficits. In order to explore whether AS-IV plays a neuroprotective role by regulating SIRT1 in ischemic brain, we inhibited SIRT1 with EX527. After intracerebroventricular injection of EX527, the AS-IV + EX527 group showed a higher NDS at 7 days after reperfusion compared with the AS-IV group (*p* < 0.05). NDS significantly increased in the MCAO/R + EX527 group when compared with the MCAO/R group at 7 days as well (*p* < 0.05) (Figure 6).

### Effects of EX527 on Infarction Area Intervened by Astragaloside IV

**FIGURE 7:**
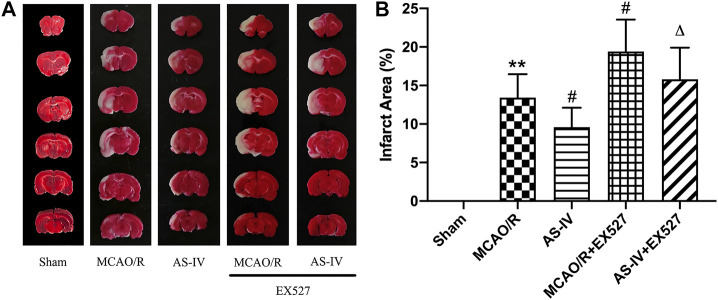
The cerebral infarction areas in Sham, MCAO/R, AS-IV, MCAO/R + EX527, and AS-IV + EX527 group at 7 days after CIR injury in rats (mean ± SD, *n* = 6). **(A)** TTC staining. **(B)** Quantitative analysis for the percentage of the infarction area. **p < 0.01, compared with the Sham group; #*p* < 0.05, compared with the MCAO/R group; ∆*p* < 0.05 compared with the AS-IV group.

After inhibiting SIRT1, the area of cerebral infarction increased at 7 days after reperfusion. Compared with the AS-IV group, the AS-IV + EX527 group showed a more severe infarction (*p* < 0.05). Similarly, compared with the MCAO/R group, the infarction area significantly increased in the MCAO/R + EX527 group at 7 days (*p* < 0.05) (Figure 7).

### Effect of EX527 on the Expression of SIRT1 Intervened by Astragaloside IV

**FIGURE 8:**
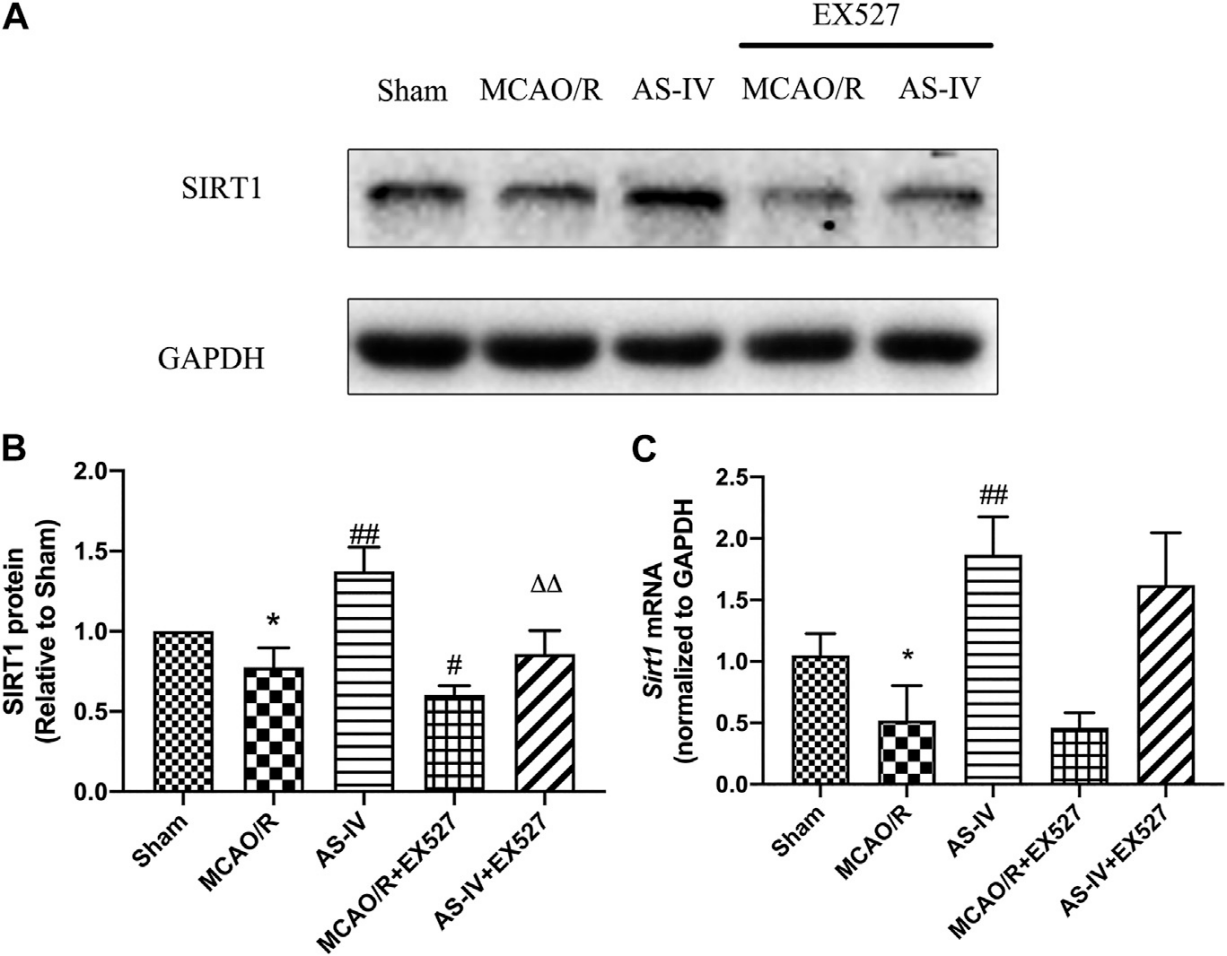
The expression of SIRT1 in Sham, MCAO/R, AS-IV, MCAO/R + EX527, and AS-IV + EX527 group at 7 days after CIR injury (mean ± SD, *n* = 6). **(A)** The results from the Western Blot. **(B)** Quantitative analysis for the protein level of SIRT1. **(C)** Quantitative analysis for the mRNA level of Sirt1. *p < 0.05, compared with the Sham group; #*p* < 0.05, ##*p* < 0.01 compared with the MCAO/R group; ∆∆*p* < 0.01 compared with the AS-IV group.

Western Blot showed that compared with the AS-IV group, the expression of SIRT1 protein significantly decreased in AS-IV + EX527 group at 7 days after reperfusion (*p* < 0.01). And comparing with the MCAO/R group, EX527 further reduced the expression of SIRT1 (*p* < 0.05) (Figures 8A,B). There were no significant differences in the expression of Sirt1 mRNA between AS-IV group and AS-IV + EX527 group, and between MCAO/R group and MCAO/R + EX527 group (Figure 8C).

### Effect of EX527 on the Expression of MAPT Intervened by Astragaloside IV

**FIGURE 9:**
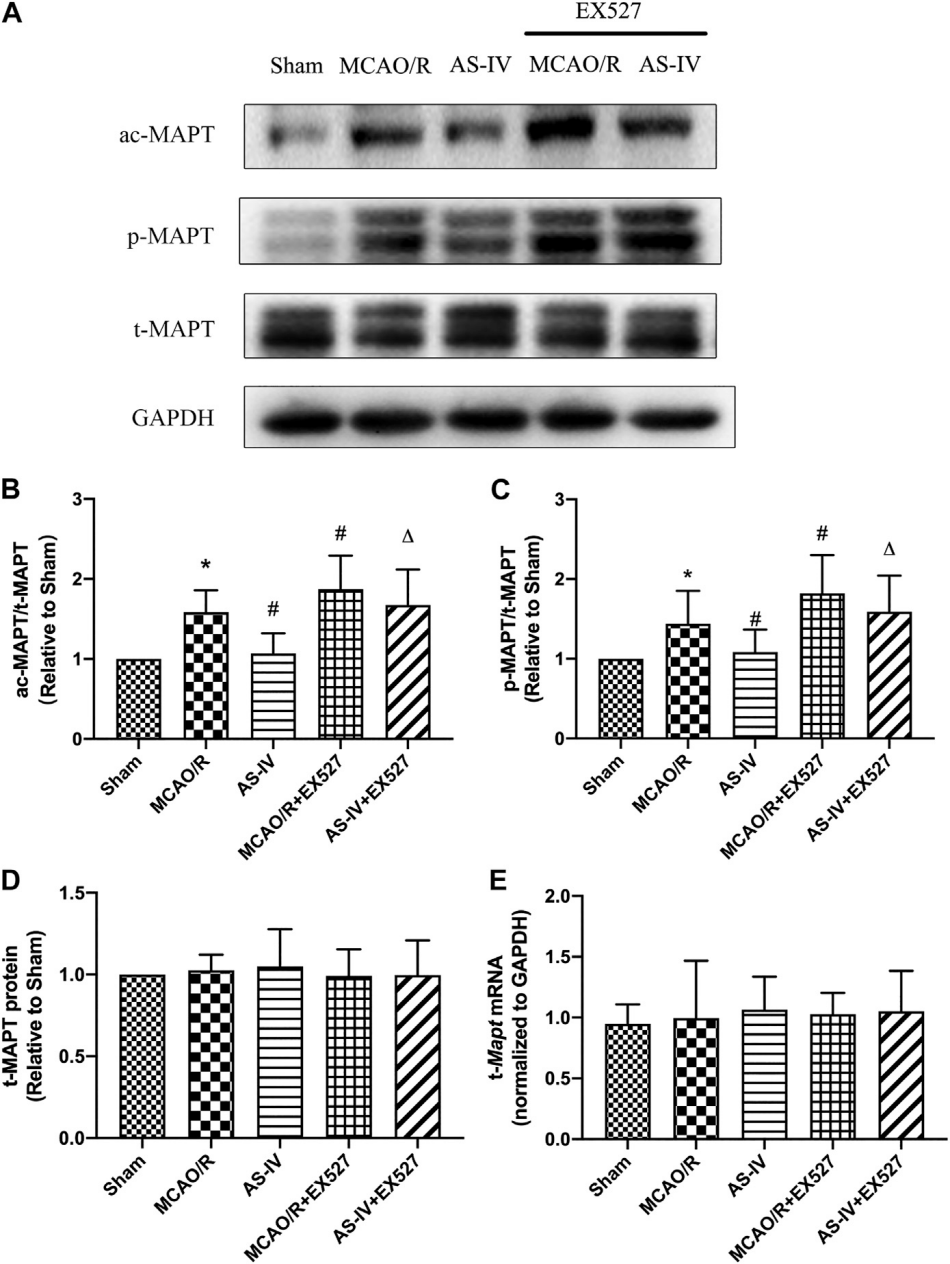
The expressions of t-MAPT, ac-MAPT, and *p*-MAPT in Sham, MCAO/R, AS-IV, MCAO/R + EX527, and AS-IV + EX527 group at 7 days after CIR injury (mean ± SD, *n* = 6). **(A)** The results from the Western Blot. **(B)** Quantitative analysis for the protein level of ac-MAPT. **(C)** Quantitative analysis for the protein level of p-MAPT. **(D)** Quantitative analysis for the protein level of t-MAPT. **(E)** Quantitative analysis for the mRNA level of t-Mapt. *p < 0.05, compared with the Sham group; #*p* < 0.05, compared with the MCAO/R group; ∆*p* < 0.05 compared with the AS-IV group.

Western Blot detected that compared with the AS-IV group, the expression of ac-MAPT significantly increased in the AS-IV + EX527 group (*p* < 0.05). Compared with the MCAO/R group, the MCAO/R + EX527 group showed a higher level of ac-MAPT (*p* < 0.05) (Figures 9A,B). The expression of *p*-MAPT in each group showed a similar trend. Compared with the AS-IV group, the AS-IV + EX527 group had a significant increase in the expression of *p*-MAPT (*p* < 0.05). While compared with the MCAO/R group, the expression of *p*-MAPT in the MCAO/R + EX527 group increased obviously (*p* < 0.05) (Figures 9A,C). Western Blot and qRT-PCR showed that both t-MAPT protein and mRNA levels were not significantly different among all groups (Figures 9A,D,E).

## Discussion

The results of this study demonstrated that AS-IV can improve neurological dysfunction, decrease infarction area, upregulate the expression of SIRT1, and decline the levels of ac-MAPT and *p*-MAPT. On the contrary, SIRT1-specific inhibitor EX527 hindered the improvements of neurological function, infarction area, and the changes of modified MAPT levels caused by AS-IV. Thus, AS-IV might exert a neuroprotective effect in the rat models of CIR injury by regulating the Sirt1/Mapt pathway.

SIRT1, belonging to the sirtuin family, is widely expressed in brain, and abundantly exits in metabolic-related areas such as the hippocampus, cortex, hypothalamus, and cerebellum (Ramadori et al., 2008). Studies have shown that SIRT1 plays an important role in ischemic stroke. Yan et al. (2013) found that CIR injury induced a down regulation of SIRT1, which was significantly different from the control group starting from 1 h after reperfusion. Moreover, overexpression or silencing of SIRT1 in CIR models significantly influenced the size of cerebral infarction area and the symptoms of neurological deficits (Koronowski et al., 2015; Ashrafi et al., 2017; Zhang et al., 2017; Zhang et al., 2018). In the present study, our results showed that the NDS and infarction area increased obviously after CIR injury. While after AS-IV intervention, both two of the outcomes declined significantly. Consistent with above findings, inhibition of SIRT1 aggravated the brain damage caused by CIR injury and abolished the protective effects of AS-IV. Moreover, the effect of AS-IV on Sirt1 in CIR brain probably is manifested as up-regulation of expression rather than enhancement of activity. Li et al. (2020) have found that, compared with the MCAO model group, Cycloastragenol (CAG, the active form of AS-IV) significantly prevented the downregulation of Sirt1 mRNA and protein levels at 1 day after MCAO, which was consistent with our findings. However, when SIRT1 activity was measured *in vitro*, CAG did not directly active the enzyme as the positive control resveratrol did. Therefore, AS-IV may enhance Sirt1 activity by upregulating its expression, exerting neuroprotective effect in the ischemic brain.

As an NAD + -dependent class III histone deacetylase, SIRT1 also catalyzes a variety of downstream substrates, playing a key role in multiple biological processes (Kane and Sinclair, 2018; Gomes et al., 2019). Zhao et al. (2019) and Teertam et al. (2019) found that overexpression of SIRT1 deacetylated p53, and thereby, inhibited its activation of downstream target genes and alleviated p53-mediated apoptosis. Sun et al. (2018) found that SIRT1 enhanced the transcription of FOXO3 through the deacetyl activity, reduced the level of reactive oxygen species in cells, and improved the ability of cells to resist oxidative stress. Recently, some studies have pointed out that MAPT may be one of the downstream targets of SIRT1 in the cytoplasm (Futch and Croft, 2018; Min et al., 2018), and inhibiting SIRT1 leaded to the accumulation of MAPT in the cell (Min et al., 2010).

As a microtubule-related cytoskeletal protein, MAPT exerts an effect of stabilizing microtubule polymerization in central nervous system mainly through the dynamic balance of post-translational modification (Caceres and Kosik, 1990). There are various types of post-translational modifications of MAPT. Many studies have pointed out that hyperacetylation of MAPT inhibits the depolymerization of itself, in other words, promotes the accumulation of abnormal MAPT in cells and results in cytotoxicity (Cohen et al., 2011; Trzeciakiewicz et al., 2017; Ferreon et al., 2018). Acetylation of lysines precludes its ubiquitination and prevented the degradation of proteins mediated by the ubiquitin-proteasome system (UPS), including p53 (Ito et al., 2002), Runx3 (Jin et al., 2004), β-catenin (Ge et al., 2009), and other regulatory factors (Caron et al., 2005). The degradation of MAPT, especially phosphorylated MAPT, is closely associated with the UPS (Petrucelli et al., 2004; Tan et al., 2008). According to the evidences provided by Min et al. (2010), acetylation of lysines 163, 174, and 180 blocks ubiquitination at these three sites and increases the half-life of MAPT. After promoting MAPT acetylation with Ex527 and inhibiting new protein translation with CHX, t-MAPT level was significantly reduced after 5 h, while ac-MAPT level was only slightly decreased after 8 h, suggesting that degradation of MAPT can be slowed by its acetylation. Besides EX527 can also block the degradation of AT8-positive *p*-MAPT in a dose-dependent manner. When inhibiting p300 (acetyltransferase) by C646, ac-MAPT was eliminated without affecting t-MAPT levels, and pathogenic *p*-MAPT was also abolished within 2 h treatment with C646, suggesting that deacetylation enhances the degradation of *p*-MAPT. The hyperphosphorylation of MAPT reduces its solubility and promotes the forming of neurofibrillary tangles, which destroys the intracellular microtubule network and ultimately leads to neuronal death (Idan-Feldman et al., 2012). Thus, targeting MAPT acetylation may be a new therapeutic strategy to reduce levels of pathogenic MAPT, especially pathogenic *p*-MAPT, in neurodegenerative tauopathies.

The role of MAPT in ischemic stroke has recently attracted wide attention. Basurto-Islas et al. (2018) found that occluding the MCA for 1 h in C57BL/6J mice, there were a large number of hyperphosphorylated MAPT (Ser262/356) that collocated with apoptotic cells. Fujii et al. (2017) established a 90 min MCAO model in Wistar rats and found that 12 h after CIR, the abnormally highly phosphorylated MAPT (Ser202/Thr205) can be detected, and the protein level reached a peak at 48 h. While, knocking out of MAPT leaded to a smaller infarction area and milder neurological deficit symptoms (Bi et al., 2017). In our study, the expression of ac-MAPT started to increase significantly at 6 h after reperfusion and was higher than that in the Sham group at all time points. AS-IV could decrease ac-MAPT level, which was negatively correlated with the trend of SIRT1. In contrast, inhibition of SIRT1 by EX527 abolished the downtrend of ac-MAPT level mediated by AS-IV, suggesting that AS-IV alleviated the hyperacetylation of MAPT in CIR rats via upregulating the expressions of SIRT1. The expression of *p*-MAPT shared a similar trend with ac-MAPT both in MCAO/R group and AS-IV group. And when intervened by EX527, the improvement effect of AS-IV was obviously weakened. Thus, AS-IV could alleviate the hyperphosphorylation of MAPT via upregulating SIRT1 and further controlling MAPT modifications.

At present, most of the studies on the physiological functions and pathological changes of MAPT are focused on the hippocampus. Previous studies have shown that hippocampal neurons are extremely sensitive to ischemia and hypoxia. 1 hour of MCAO (Ward et al., 2018) or global cerebral ischemia for 5 min (Lee et al., 2017) can lead to cell death and neurodegeneration. Luo et al. Luo et al. (2018) found that in mice with MCAO for 90 min, the expression of *α*2*δ*−1 significantly increased 24 h after CIR injury, which activated N-methyl-D-aspartate receptors, promoted the expression of Cleaved-caspase-3, and then poisoned neurons. Thus, the change of perfusion caused by distal artery occlusion can also have a great impact on hippocampal neuron. Besides, although hippocampus is related to the function of cognition and memory, there is often a delay in the development of cognitive decline after ischemic stroke onset (Brainin et al., 2015), while the motorial and sensory disorders occurs immediately. Because of the neurological function and infarction area are two of the essential outcomes in the preclinical assessment of stroke therapy (Fisher et al., 2009), we selected them as the indicators to evaluate the neuroprotective effects of AS-IV.

The present study still has some limitations. In our study, we mainly investigated the relationship among AS-IV, SIRT1 and the acetylation/phosphorylation status of MAPT, without further research into the interaction mechanisms between different post-translational modifications of MAPT, which is our future research directions as well.

## Conclusion

The present study demonstrated that AS-IV can improve neurological deficits and alleviate cerebral infarction area in rats with CIR injury. AS-IV exerted the neuroprotective effects against the damage of cerebral ischemia probably by the activation of Sirt1/Mapt pathway.

## Data Availability

The original contributions presented in the study are included in the article/Supplementary Material, further inquiries can be directed to the corresponding authors.

## Abbreviations

rtPA, recombinant tissue plasminogen activator; SIRT1, silent information regulator 1; MAPT, microtubule-associated protein tau; CIR, cerebral ischemia/reperfusion; SD, sprague–dawley; MCAO/R, middle cerebral artery occlusion/reperfusion; CCA, common carotid artery; ICA, internal carotid artery; ECA, external carotid artery; mNSS, modified neurological severity score; TTC, triphenyl tetrazolium chloride.
